# Impact of SliP4 deletion on the high-light acclimation in *Synechocystis* sp. PCC 6803

**DOI:** 10.1093/femsml/uqag003

**Published:** 2026-01-19

**Authors:** Luna Alvarenga-Lucius, Sandra Maaß, Viktoria Reimann, Dörte Becher, Wolfgang R Hess, Martin Hagemann

**Affiliations:** Institute of Biosciences, Department of Plant Physiology, University of Rostock, D-18059 Rostock, Germany; Institute of Microbiology, Department of Microbial Proteomics, University of Greifswald, D-17489 Greifswald, Germany; University of Freiburg, Faculty of Biology, Genetics and Experimental Bioinformatics, Schänzlestr. 1, D-79104 Freiburg, Germany; Institute of Microbiology, Department of Microbial Proteomics, University of Greifswald, D-17489 Greifswald, Germany; University of Freiburg, Faculty of Biology, Genetics and Experimental Bioinformatics, Schänzlestr. 1, D-79104 Freiburg, Germany; Institute of Biosciences, Department of Plant Physiology, University of Rostock, D-18059 Rostock, Germany

**Keywords:** Gene expression, proteome, metabolome, high light, photosynthesis, small proteins

## Abstract

SliP4 is a small, 37 amino acids protein that is strongly induced when the cyanobacterium *Synechocystis* sp. PCC 6803 is exposed to high-light (HL) conditions. Deletion mutants manifest a light-sensitive phenotype due to impaired cyclic electron flow and state transitions. In this study, we aimed to investigate the consequences of SliP4 deficiency on the process of high-light acclimation on systems level. Transcriptomic data revealed that the deletion mutant Δ*sliP4* exhibited a wild-type-like gene regulatory response 30 minutes after the light intensity was increased from 50 to 250 μmol photons m^−2^ s^−1^, a process that is controlled by the RpaB-PsrR1 system. Proteome analysis showed consistent expression changes of many HL-regulated proteins. Metabolome analysis provided hints for a changed N and C metabolism in mutant cells compared to wild type. In addition, the mutant increased the production of extracellular polysaccharides causing the mutant cells to aggregate after the shift to HL. This effect corresponds to the upregulated expression of *xssA-E* and *xssN-P* genes for the production of the sulfated exopolysaccharide synechan. We interpret these observations as a response that counteracts the potential light stress effects caused by the impaired capacity for cyclic electron flow and state transitions in the Δ*sliP4* mutant. Our results demonstrate that the unicellular cyanobacterium *Synechocystis* compensates for the loss of SliP4 and its crucial role by activating a genetic program for a population-level response that helps the cells to cope with HL conditions.

## Teaser

The lack of the small high-light-induced protein SliP4 has a marked impact on the acclimation response of *Synechocystis* sp. PCC 6803 to high light.

## Introduction

Oxygenic photosynthesis is the most important biochemical process driving the carbon cycle on a global scale. Oxygenic phototrophs, such as cyanobacteria, algae, and plants, use light energy from the sun to convert inorganic CO_2_ into organic compounds, releasing molecular O_2_ as a byproduct of water splitting. Hence, all oxygenic phototrophs depend on light as an energy source. However, excess light can also have detrimental effects. The exposure of oxygenic phototrophs to intense light is typically accompanied by redox imbalances, resulting in the production of various redox-active compounds, mainly reactive oxygen species (ROS). The initial target of high light (HL) stress is the photosystem II (PSII) subunit D1 (PsbA), which is quickly destroyed in the presence of HL-induced ROS and must be replaced by an intact version in the PSII repair cycle (e.g. Johnson and Pakrasi 2022). The appearance of ROS or redox imbalances in the photosynthetic electron transport chain induces a complex acclimation reaction to cope with HL intensities and combat ROS. A primary strategy employed to mitigate excess light is the dissipation of energy as heat, a process known as non-photochemical quenching (NPQ). This is accompanied by cyclic electron flow (CEF), state transition, oxygen photoreduction, and photorespiration (discussed in more detail by Dann et al. (2021)).

The acclimation to HL has been intensively investigated during the last decades in different model systems. Among cyanobacteria, *Synechocystis* sp. PCC 6803 (hereafter *Synechocystis*) is widely used as a model in photosynthesis research due to its genetic tractability and, particularly, due to its ability to grow in the presence of glucose, which permits the knockout of genes for essential photosynthetic subunits (Grigorieva and Shestakov 1982, Koksharova and Wolk 2002). Like other oxygenic photosynthetic organisms, *Synechocystis* is highly susceptible to fluctuations in environmental light conditions. Early investigations of HL-induced transcriptomic changes revealed that hundreds of the 3 700 genes in the *Synechocystis* genome became induced or repressed under these stress conditions (Hihara et al. 2001). That work and following studies identified many genes that encode proteins for various photoprotective strategies to mitigate the detrimental effects caused by HL. One class of photo-protective proteins consists of the small HL-inducible proteins (HLIPs), which often reach very high expression levels during the acclimation response. HLIPs bind chlorophyll molecules associated with carotenoids and therefore help to emit excess absorbed photons (Tibiletti et al. 2018, Konert et al. 2022). Another important player is the orange carotenoid protein (OCP), which induces thermal dissipation of excess excitation energy by interacting with the phycobilisome, the main mechanism of NPQ in *Synechocystis* and other cyanobacteria (Kirilovsky and Kerfeld 2012, 2016). In another mechanism, different sets of flavodiiron proteins are involved in energy dissipation via O_2_ photoreduction, i.e. Mehler-like reactions among cyanobacteria (Helman et al. 2003, Nikkanen et al. 2025). Last but not least, the entire electron transport chain becomes organized in different modes optimizing the ratio of linear electron flow and CEF (e.g. Theune et al. 2021).

The acclimation to HL is regulated on different layers. An important transcription factor for the acclimation to different light intensities is RpaB (“regulator of phycobilisome association B”) (Ashby and Mullineaux 1999). Its cognate histidine kinase Hik33 responds not only to light intensity (Tu et al. 2004), but also to low temperature (Suzuki et al. 2000), hyperosmolarity (Mikami et al. 2002), high salinity (Marin et al. 2003), oxidative stress (Kanesaki et al. 2007), and nutrient limitation (van Waasbergen et al. 2002). In *Synechocystis*, RpaB controls the transcription from at least 167 promoters of protein-coding genes and operons as well as 22 promoters of regulatory non-coding RNAs (Riediger et al. 2019). Among the controlled genes are *hliA* and *hliB* (Kappell and van Waasbergen 2007), several genes encoding PSI proteins (Seino et al. 2009, Takahashi et al. 2010), CRISPR2-Cas system genes (Bilger et al. 2024), the small RNA (sRNA) gene *psrR1* (Kadowaki et al. 2016), six out of nine ferredoxin genes (Riediger et al. 2019), and *pgr5/ssr2016* encoding a small protein involved in CEF from PSI to the PQ pool (Yeremenko et al. 2005). RpaB can activate as well as repress transcription, depending on the location of the binding site within the promoter (Wilde and Hihara 2016, Riediger et al. 2018). Therefore, it can control the decrease in the transcript levels of PSI genes upon shifts to HL, while it can induce the transcription of other genes such as *hli* and *psrR1* under the same condition (Kadowaki et al. 2016). PsrR1 is a regulatory sRNA that functions as a negative post-transcriptional regulator of genes encoding phycobiliproteins and PSI proteins (Georg et al. 2014). Therefore, the concerted action by RpaB and PsrR1 leads to the dual repression of PSI genes under HL at the transcriptional and post-transcriptional level (Kadowaki et al. 2016).

In recent years, a plethora of small proteins (≤ 80 amino acids) have been identified in cyanobacteria (Brandenburg and Klähn 2020, Kraus and Hess 2024). Many of them play structural roles in photosynthetic complexes (see Baumgartner et al. 2016). However, there are also many examples that they can regulate photosynthetic or metabolic processes, such as AtpΘ inhibiting ATP synthase activity under low energy conditions (Song et al. 2022), NblD required for the degradation of phycobilisomes during nitrogen starvation (Krauspe et al. 2021), AcnSP regulating aconitase activity (deAlvarenga et al. 2020), or NirP1, an inhibitor of nitrite reductase activity under low carbon conditions (Kraus et al. 2024). Recently, we showed that the **s**mall **l**ight-**i**nduced **p**rotein of **4** kDa, SliP4 (initially called *hliR1* in Baumgartner et al. 2016), plays an essential role in acclimation of *Synechocystis* to HL conditions (Alvarenga-Lucius et al. 2023). The expression of SliP4 is controlled by RpaB (Riediger et al. 2019) and increases sharply when the light intensity is elevated. SliP4 was then found to play a pivotal role in facilitating the association of the NDH1 complex involved in CEF with either PSI or PSII, thereby enabling a variety of electron transfer mechanisms within thylakoid membranes, particularly under HL conditions. Moreover, the knockout of *sliP4* resulted in a mutant that has difficulties acclimating to switches from growth light to HL, i.e. from 50 to 250 μmol photons m^−2^ s^−1^ (Alvarenga-Lucius et al. 2023).

In the present study, we investigated the system-wide effect of SliP4 deficiency on the HL acclimation in *Synechocystis*. For this purpose, we combined different “omics” methods to quantify metabolomic and gene expression changes on the mRNA and protein levels before and after HL treatment. The results revealed that the lack of SliP4 triggered alternative measures to deal with HL, including the induction of extracellular polysaccharide (EPS) synthesis to avoid light stress via cell aggregation, a strategy that has been identified recently to lead to higher light tolerance in *Synechocystis* using adaptive laboratory evolution (Dann et al. 2021).

## Materials and methods

### Culture conditions

The *Synechocystis* sp. PCC 6803 substrain M (Trautmann et al. 2012) was used throughout. The generation of the ∆*sliP4* mutant was described in detail by Alvarenga-Lucius et al. (2023). The wild-type (WT) and ∆*sliP4* mutant strains were grown in glass Erlenmeyer flasks (125 mL) filled with 40 mL of BG-11 buffered medium (pH 8.0) without sodium carbonate (Rippka et al. 1979) under the following conditions: 28 ± 2°C, continuous light of 50 μmol photons m^−2^ s^−1^. Growth experiments were performed for 48 h in a Multi-Cultivator MC1000-OD at 30°C (Photon Systems Instruments, Czech Republic) with continuous illumination of 250 μmol photons m^−2^ s^−1^ and bubbling with 5% (v/v) CO_2_ supplemented. The ∆*sliP4* mutant was grown in the presence of 50 µg mL^−1^ kanamycin during pre-culture and without antibiotics during the experiments for accurate comparison with the WT. All experiments except the microarray hybridizations were performed with three biological and two technical replicates.

### RNA extraction

Cells were collected by centrifugation at 7 000 *x* g for 5 min at 4°C. RNA was extracted using the hot phenol method as described (Hein et al. 2013) with the modification that cells were first pelleted and frozen in liquid nitrogen. Then, PGTX buffer (Pinto et al. 2009) was added to the frozen cell pellets. Samples were incubated for 15 min at 65°C in a water bath. After the addition of 1 volume chloroform: isoamyl alcohol (24:1), they were incubated for 15 min at room temperature with several vortexing cycles, followed by precipitation with an equal volume of isopropanol.

### Transcriptome analysis

For transcriptome analysis, custom-made Agilent one-color microarrays were used that allow the direct detection of transcripts after chemical labelling (Voß and Hess 2014). The microarray analyses were performed in two technical replicates. The microarrays contain oligonucleotide probes for all annotated mRNAs and the majority of non-coding RNAs. Total RNA (5 µg) was extracted from the WT and ∆*sliP4* mutant grown at 50 μmol photons m^−2^ s^−1^ and after a shift to 250 μmol photons m^−2^ s^−1^ for 30 min and 48 h, respectively. Total RNA was DNase-treated using TURBO DNase (Thermo Fisher Scientific). 2 µg DNase-treated RNA were directly labeled with Cy3 using the ULS-Fluorescent Labeling Kit for Agilent gene expression arrays as recently described (Hemm et al. 2025). A high-resolution microarray manufactured by Agilent (Design ID 075764, format 8 × 60 K, slide Layout = IS-62976–8-V2) was used. 600 ng labeled RNA were used for hybridization for 17 h at 65°C, per array. Raw data were normexp background corrected (Ritchie et al. 2015) and quantile normalized using the limma R package (Ritchie et al. 2007). The transcriptome differences were calculated between WT and ∆*sliP4* mutant for the two different light conditions and time points. Significance criteria for differential expression were log_2_FC ≥│1│, adj. p -value ≤ 0.05. *P* values were adjusted for multiple testing with the Benjamini-Hochberg method. The microarray data has been deposited in the GEO database under the accession number GSE302282.

### Proteome analysis

Cell aliquots of 10 mL were harvested by centrifugation after 0 and 48 h of growth under high light and extracted by two cycles of sonication. Proteins were separated by standard SDS-PAGE (12% acrylamide) using the minigel system (Bio-Rad). Proteins were denatured by 5 min boiling in Laemmli-buffer containing 70 mM SDS and 2.4 mM 2-mercaptoethanol. The separated proteins were either stained with Coomassie Brilliant Blue. Gel lanes were excised and cut into ten equidistant pieces. In-gel protein digest was performed as described by Bonn et al. (2014). Briefly, gel pieces were de-stained and dried in a vacuum centrifuge before proteins were in-gel digested with trypsin. The resulting peptides were eluted and subjected to mass spectrometry. LC–MS/MS analyses were performed with an EASY-nLC 1000 liquid chromatography system coupled to a LTQ Orbitrap Velos (Thermo Fisher Scientific, Waltham, MA, USA) as described previously by Alvarenga-Lucius et al. (2023).

Resulting raw files were searched with MAXQUANT (v.2.1.3.0) against a database of *Synechocystis* downloaded from Uniprot on 18/12/2019 and supplemented with sequences of Flag-tagged, nonannotated small proteins (3510 entries in total). Common laboratory contaminants and reversed sequences were included by MAXQUANT. Search parameters were set as follows: Trypsin/P specific digestion with up to two missed cleavages, methionine oxidation, and N-terminal acetylation as variable modifications. The FDRs (false discovery rates) of protein and PSM (peptide spectrum match) levels were set to 0.01. The MS data have been deposited in the ProteomeXchange Consortium via the PRIDE partner repository (Vizcaino et al. 2016) with the dataset identifier PXD065318.

### Metabolome analysis

Cell aliquots of 5 mL were harvested by filtration after 0 or 24 h of growth under HL, and metabolites were cold-extracted using a 2:1:2 methanol: chloroform: H_2_O mixture (LC-MS Grade, Roth, Germany). One microgram of carnitine was added to each sample as an internal standard. After centrifugation, the supernatants were collected and lyophilised. The dry extracts were then dissolved in 200 µL of MS-grade water and filtered through 0.2 µm filters (Omnifix®-F, Braun, Germany). The clarified supernatants were analysed using a high-performance liquid chromatograph mass spectrometer system (LCMS-8050, Shimadzu, Japan). Briefly, 1 µL of each extract was separated on a pentafluorophenylpropyl (PFPP) column (Supelco Discovery HS FS, 3 µm, 150×2.1 mm) with a mobile phase containing 0.1% formic acid. Compounds were eluted at a rate of 0.25 mL/min using the following gradient: 1 min at 0.1% formic acid in 5% acetonitrile and completed with distilled water; followed by a linear gradient over 15 min to 0.1% formic acid, 5% distilled water, 95% acetonitrile; and finally, 10 min at 0.1% formic acid, 5% distilled water, 95% acetonitrile. Aliquots were continuously injected into the MS/MS section and ionised by electrospray ionisation (ESI). Compounds were identified and quantified using the multiple reaction monitoring (MRM) values provided in the LC-MS/MS method package and LabSolutions software package (Shimadzu, Japan). Metabolites were determined as relative metabolite abundances, calculated by normalising signal intensity to that of the internal standard carnitine and their respective OD_750_ mL^−1^.

### Soluble and insoluble carbohydrates

Total soluble carbohydrates and Released Exopolysaccharides (RPS) present in the medium from cells exposed to 48 h of HL were quantified. The Capsular Exopolysaccharides (CPS) and total soluble carbohydrates were first released from the cells with 0.005% NaCl solution and incubated for 30 min at 60°C, followed by 10 min in an ultrasonic bath (Strieth et al. 2020). The samples were then centrifuged, and the supernatants were used for quantification with the reagent o-toluidine (Sigma Aldrich) at 635 nm in a microplate reader. The pellet was washed with ethanol, treated with amyloglucosidase (ROAMYGL, Roche) for 2 h at 60°C and later used for glycogen quantification as above. All the samples were normalized by a glucose standard curve to their respective OD_750_ values and the total volume of the initial sample.

The floating test was reproducibly done using the two-step culture regime developed by Maeda et al. (2021). In the first step, cells inoculated at OD_730_ = 0.2 were grown with 5% CO_2_ aeration under continuous light of 250 μmol photons m^−2^ s^−1^ for 24 h. In the second step, the culture was shifted to standing conditions without bubbling under 10 μmol photons m^−2^ s^−1^ light for another 24 h for the cells to rise to the surface.

## Results

To analyse the overall impact of SliP4 on HL acclimation, we first grew the *Synechocystis* WT and mutant ∆*sliP4* under a light intensity of 50 μmol photons m^−2^ s^−1^ and then transferred the cells to 250 μmol photons m^−2^ s^−1^ for up to 48 h. Identical treatments were used in the previous study in which the physiological roles of SliP4 during the HL acclimation were investigated in detail (Alvarenga-Lucius et al. 2023). Here, we combined transcriptome, proteome and metabolome techniques to obtain a system-wide overview of the HL acclimation changes in the mutant ∆*sliP4* compared to WT. The growth response of the strains to HL treatment and 236 the sampling points are displayed in the Supplementary Fig. S1. The functional importance of altered expression of enzymes for a specific group of EPS was verified in physiological experiments.

### Transcriptome analysis identifies a small number of gene expression changes in the ∆sliP4 mutant

**Figure 1:**
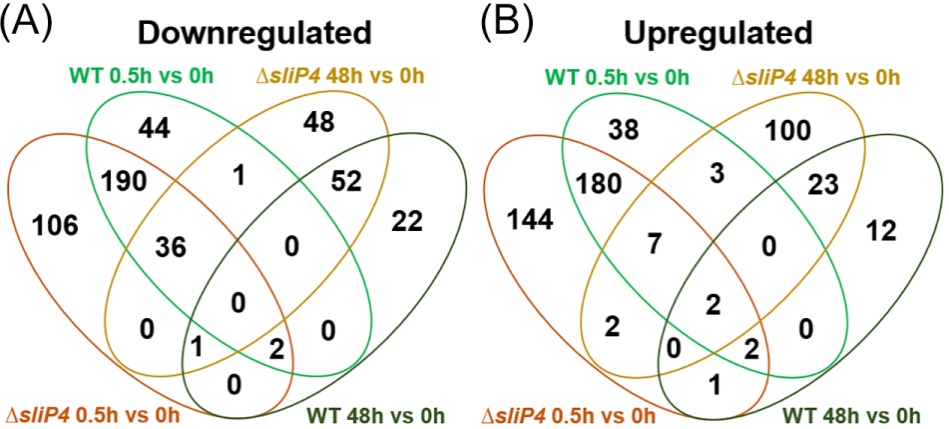
Comparison of differentially expressed transcripts. A. Comparison of all down-regulated transcripts in WT and *∆sliP4* after 0.5 h and 48 h at HL compared to T_0_. Filtered for log_2_FC ≥│1│, p_adj_ ≤ 0.05 (N = 823). B. Comparison of all upregulated transcripts in WT and *∆sliP4* after 0.5 h and 48 h at HL compared to T_0_. Filtered for log_2_FC ≥│1│, p_adj_ ≤ 0.05 (N = 517).

Gene expression analyses were performed before and 30 min or 48 h after exposure to HL (the entire data set can be found in Supplementary Table S1 and Supplementary Table S2). Our direct RNA labelling approach together with the design of the used microarrays allow the detection of expression changes not only of annotated genes, but of all 9 234 transcriptomic features, including non-coding RNAs (sRNAs and antisense RNAs), 5’ and 3’ UTRs previously defined on the basis of comparative analyses of the primary transcriptome (Kopf et al. 2014). Gene expression changes ≥ log_2_ of │1│ at a *p* value ≤ 0.05 were regarded as significant. A quantitative comparison showed that approximately 500 transcripts showed higher expression in the two strains upon HL treatment for 0.5 or 48 h, while more than 800 transcripts were expressed at lower levels (Fig. 1). Among them, more than 50% of the upregulated genes are shared between cells of the WT and the mutant ∆*sliP4* after the short-term HL shift.

Many genes, especially those encoding proteins with assigned functions to acclimate towards HL stress showed a similar response to the HL treatment in the ∆*sliP4* mutant compared to WT (Table 1). The most significant alterations in expression were observed for the HLIP-encoding genes *hliA* (*ssl2542*), *hliB* (*ssr2595*), and *hliC* (*ssl1633*), which were highly expressed after 30 min of HL in the WT and in ∆*sliP4*. Moreover, both strains displayed an increased expression of genes encoding nitrate and ammonia uptake and assimilation systems, while the expression of the phycobilisome antennae was strongly decreased 30 min after HL exposure. Only a few photosynthesis-associated genes such as *psbA3* encoding the D1 protein (*sll1867*), two NDH1 subunits (*ndhB, sll0233*, and *ndhE, sll0522*) and ferredoxin 1 (*petF, ssl0020*) showed increased expression following 30 min of exposure to 250 μmol photons m^−2^ s^−1^ (Table 1), whereas the majority of genes encoding subunits of the PSII and NDH1 complexes did not show significant expression changes (Supplementary Table S1). In addition to phycobilisome subunits, several genes encoding PSI subunits (*slr1655, slr1834, slr1835, ssl0563* and *smr0004*), and genes associated with the CRISPR2-Cas system showed significantly decreased expression. Finally, genes encoding regulatory proteins or sRNAs such as *glnB* (PII, *ssl0707*), PmgR1 (*ncr0700*) and PsrR1 (*ncr06900*) showed altered gene expression (Table 1). The sRNA pair PmgR1/PsrR1 showed an inverse regulation, with a strong upregulation of PsrR1, an important acclimation response to HL (Georg et al. 2014), mediated by the transcription factor RpaB (Kadowaki et al. 2016). The observed log_2_FC of +3.7 for PsrR1 (Table 1) corresponds well to published induction levels measured by other methods. An approximately 16-fold induction was previously determined in a shift from 50 to 300 µmol photons m^−2^ s^−1^ by Northern hybridization (see Fig. 2 in Georg et al. 2014), indicating an adequate dynamic range and a good reproducibility of our analysis.

**Figure 2:**
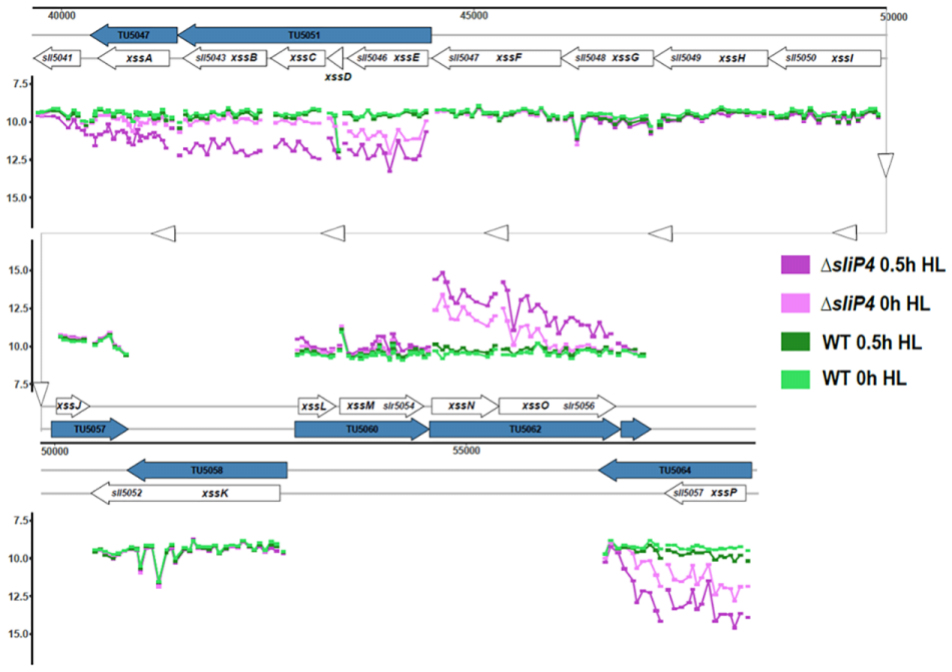
Gene expression changes in the synechan gene cluster. The genomic region from position 39 650 to 57 950 on plasmid pSYSM is shown encompassing the synechan-related genes *xssA* to *xssP* (locus IDs *slr5042* to *sll5057*) (Maeda et al. 2021). Microarray probes are visualized by short horizontal tabs connected by coloured lines according to the enclosed colour scheme. For better orientation, also some locus IDs are given and transcriptional units (TU) according to previous dRNA-Seq mapping of transcriptional start sites and transcripts (Kopf et al. 2014). Note that the lower panel is a continuation of the upper panel, from position 50 000. For genes *xssF* to *xssM*, only baseline expression was detected without any regulation between the conditions and strains.

**Table 1:** Transcriptomics after short-term high light (HL) treatment.

gene	gene description	∆*sliP4* 0.5 h vs 0h	WT 0.5 h vs 0h	∆*sliP4* 0 h vs WT 0h	∆*sliP4* 0.5 h vs WT 0.5h
**sll5057**	probable glycosyltransferase (xssN)	1.80	0.78	3.43	4.45
**sll5046**	synechan system (xssE)	1.29	0.01	2.41	3.68
**sll5044**	synechan system (xssC)	2.42	0.02	1.15	3.55
**sll5043**	probable glycosyltransferase (xssB)	2.66	0.17	0.91	3.40
**slr5056**	probable glycosyltransferase (xssO)	2.09	0.13	1.29	3.26
**sll5042**	probable sulfotransferase (xssA)	1.54	-0.03	0.85	2.42
**slr1667**	hypothetical protein (target gene of sycrp1)	-4.41	-0.90	4.99	1.48
**sll1451**	nitrate/nitrite transport system permease (nrtB)	3.75	1.91	-0.44	1.39
**sll1452**	nitrate/nitrite transport system ATP-binding (nrtC)	4.16	2.09	-0.77	1.31
**sll1450**	nitrate/nitrite transport system substrate-binding (nrtA)	3.27	1.79	-0.23	1.25
**sll1453**	nitrate/nitrite transport system ATP-binding (nrtD)	4.44	2.37	-0.97	1.11
**ssl0453**	phycobilisome degradation protein (nblA)	2.65	2.04	0.37	0.98
**ssr2016**	PGR5 homolog involved in cyclic electron transport	3.84	2.90	0.03	0.97
**sll0108**	putative ammonium transporter	4.47	2.41	-1.19	0.86
**slr1200**	urea transport system permease protein	2.87	1.39	-0.78	0.70
**slr0898**	ferredoxin–nitrite reductase (nirA)	2.92	1.85	-0.41	0.67
**ssr2595**	high light-inducible polypeptide B (hliB)	3.96	3.84	0.55	0.67
**sll0223**	NAD(P)H-quinone oxidoreductase subunit 2 (ndhB)	1.14	0.99	0.39	0.54
**sll0522**	NAD(P)H-quinone oxidoreductase subunit 4 L E (ndhE)	1.05	0.71	0.16	0.50
**ssl2542**	high light inducible protein A (hliA)	4.10	4.09	0.46	0.47
**sll7067**	CRISPR2-Cas (cas10)	-1.58	-0.99	0.76	0.17
**slr1668**	function unknown (target gene of sycrp1)	-2.48	-0.12	2.47	0.11
**sll7065**	CRISPR2-Cas (cas7-2x)	-1.96	-0.97	1.09	0.10
**sll7064**	CRISPR2-Cas (csx19)	-2.10	-0.95	1.23	0.09
**sll1867**	PSII D1 protein (psbA3)	1.13	1.10	0.03	0.07
**ssl1633**	high light-inducible polypeptide C (hliC)	4.07	4.28	0.24	0.03
**ssl0707**	nitrogen regulatory protein P-II (glnB)	1.96	1.54	-0.40	0.02
**ncr0700**	sRNA pmgR1	-4.13	-3.55	0.60	0.01
**ncr0690**	sRNA psrR1	3.74	3.76	-0.01	-0.02
**sll7066**	CRISPR2-Cas (cas7-cas5-cas11)	-2.22	-1.00	1.19	-0.03
**sll7063**	CRISPR2-Cas (cas7 with insertion)	-2.26	-0.88	1.28	-0.10
**sll7062**	CRISPR2-Cas (csm6)	-1.47	-0.45	0.92	-0.10
**slr2051**	phycobilisome rod-core linker polypeptide (cpcG)	-1.83	-1.73	-0.30	-0.39
**ssl0020**	ferredoxin 1 (petF)	1.40	1.99	0.10	-0.49
**sml0002**	PSII reaction center protein X (psbX)	-1.27	-1.10	-0.33	-0.51
**slr1655**	PSI reaction center subunit XI (psaL)	-2.33	-2.33	-0.52	-0.52
**slr2067**	allophycocyanin alpha subunit (apcA)	-1.73	-1.80	-0.63	-0.55
**ssl0563**	PSI iron-sulfur center (psaC)	-1.85	-1.88	-0.63	-0.60
**smr0004**	PSI subunit VIII (psaI)	-2.34	-2.17	-0.43	-0.60
**sll1577**	phycocyanin beta subunit (cpcB)	-2.04	-2.41	-0.99	-0.62
**slr1834**	PSI P700 chlorophyll a apoprotein A1 (psaA)	-2.94	-2.36	-0.09	-0.67
**slr1986**	allophycocyanin beta subunit (apcB)	-1.88	-1.93	-0.73	-0.68
**ssr3383**	phycobilisome small core linker polypeptide (apcC)	-2.47	-2.32	-0.54	-0.69
**ssl3093**	phycobilisome small rod linker polypeptide (cpcD)	-3.16	-3.30	-0.84	-0.69
**slr1835**	PSI P700 chlorophyll a apoprotein A2 (psaB)	-2.96	-2.31	-0.07	-0.72
**sll1580**	phycobilisome 32.1 kDa linker polypeptide, rod 1 (cpcC1)	-3.84	-3.71	-0.74	-0.87
**sll1579**	phycobilisome 32.1 kDa linker polypeptide, rod 2 (cpcC2)	-4.04	-3.81	-0.77	-1.00

Gene expression changes of selected genes were compared in cells of the of *Synechocystis* WT and the ∆*sliP4* mutant after 30 min of HL (250 μmol photons m^−2^ s^−1^) exposure compared to gene expression at growth light (0 h, 50 μmol photons m^−2^ s^−1^). The locus IDs are given according to previous work (Kaneko et al. 1996, Kaneko et al. 2003), followed by the description of the gene products and four columns with the respective pairwise gene expression changes (log_2_FC) as indicated. The annotations of synechan system genes are according to Maeda et al. (2021), and the annotation of CRISPR2-Cas system genes according to Bilger et al. (2024).

Then we screened the dataset for genes that showed an altered HL response in the ∆*sliP4* mutant compared to WT. Notably, the genes for phycobilisome and PSI subunits showed a tendency to be more strongly down-regulated in the mutant, an effect which was already observed before the stress treatment (Table 1). Furthermore, several genes for nitrogen transporters (*sll0108, sll1450, sll1451, sll1452*, and *sll1453*), as well as the gene for the nitrogen regulatory protein PII (*ssl0707*), were less expressed in cells of the mutant already at growth light (time 0 h). In both strains, these genes exhibited increases after exposure to HL for 30 min. However, the expression of the nitrogen transport system was increased up to 20-fold in the mutant and only up to 5-fold in the WT (Table 1). Furthermore, the expression level of the CRISPR2-Cas system genes *sll7062, sll7063, sll7064, sll7065, sll7066*, and *sll7067* encoding all the proteins of this CRISPR2-Cas effector complex was upregulated in the mutant already before the HL treatment, while they were down-regulated by up to 4.5 times in the mutant and by up to 2 times in the WT after HL exposure.

Interestingly, the most unique expression pattern was identified for genes of the synechan system (*sll5042, sll5043, sll5044, sll5046, sll5057, slr5055, slr5056*), which encodes proteins for the synthesis of a sulfated EPS. These genes were highly induced at time 0 h in the mutant and had their expression further increased, up to 5 times, after 0.5 h under HL (Table 1). It is noteworthy, though, that not the entire synechan gene cluster was upregulated, but only the genes *sll5042* to *sll5046* and *slr5055* to *sll5057* (Fig. 2) encoding two sulfotransferases and five glycosyltransferases. These genes were previously renamed to *xssA-E* and *xssN-P* and reported to be jointly regulated by the sensory histidine kinase XssS, the response regulator XssR, and the XssQ transcriptional regulator (Maeda et al. 2021). We conclude that the observed upregulation of synechan genes most likely have occurred through the XssS/XssR/XssQ system.

After 48 h of HL, less genes showed changed expression levels compared to those observed for short-term light stress (Fig. 1). However, there was still some overlap in the long-term HL response between WT and ∆*sliP4* mutant. The mRNA level for HliC remained higher in both strains, while that for the HliB protein was only elevated in WT cells. Consistent with the less-pigmented phenotype of the ∆*sliP4* mutant (see Alvarenga-Lucius et al. 2023), many genes for phycobilisome subunits and some for the PSI and PSII were clearly more downregulated in mutant than in WT cells (Table 2). In addition, several genes encoding ATP synthase subunits were less expressed in the mutant. The genes associated with nitrate uptake were still less expressed in the mutant, while their expression increased again in the WT (Table 2). The mRNA level for ferredoxin 2 (*sll1382*), which is essential in *Synechocystis* and is highly conserved between cyanobacteria, algae, and higher plants (Schorsch et al. 2018), was elevated, whereas that for plastocyanin (*sll0199*) was lowered in mutant cells. In contrast to the short-term HL treatment, the expression of the synechan and CRISPR2-Cas system gene clusters was not further significantly different between the mutant and WT after 48 h of HL exposure.

**Table 2:** Transcriptomics after long-term high light (HL) treatment.

gene	gene description	∆*sliP4* 48 h vs 0h	WT 48 h vs 0h	∆*sliP4* 0 h vs WT 0h	∆*sliP4* 48 h vs WT 48h
**ssl1633**	high light-inducible polypeptide C (hliC)	2.70	0.93	0.26	2.03
**ssl0707**	nitrogen regulatory protein P-II (glnB)	1.43	0.27	-0.38	0.77
**slr1516**	superoxide dismutase (sodB)	1.40	0.43	-0.18	0.79
**sml0003**	PSII reaction center M (psbM)	1.18	0.12	-0.60	0.45
**sll1382**	ferredoxin, petF-like protein	0.89	0.10	-0.15	0.64
**ssr1480**	RNA-binding protein (rpb2)	0.89	-0.52	0.02	1.42
**sml0007**	photosystem II protein Y (psbY)	0.81	0.25	0.02	0.58
**ssl0020**	ferredoxin I (petF)	0.79	-0.20	-0.57	0.41
**ssl2542**	high light inducible protein A (hliA)	0.60	0.46	0.75	0.90
**sll1452**	nitrate/nitrite transport system ATP-binding (nrtC)	0.12	0.85	-0.35	-1.08
**sll1451**	nitrate/nitrite transport system permease (nrtB)	0.05	1.03	-0.07	-1.05
**sll1450**	nitrate/nitrite transport system substrate-binding (nrtA)	0.03	0.76	-0.15	-0.88
**slr0898**	ferredoxin–nitrite reductase (nirA)	0.00	0.61	0.25	-0.36
**sll0517**	RNA-binding protein (rpbA/rpb1)	-0.08	-1.31	0.12	1.34
**ssr2595**	high light-inducible polypeptide B (hliB)	-0.09	0.91	0.19	-0.81
**ssl0563**	PSI subunit VII (psaC)	-0.30	0.44	-0.38	-1.12
**slr0737**	PSI subunit II (psaD)	-0.39	0.09	-0.72	-1.20
**slr0343**	cytochrome b6-f complex subunit 4 (petD)	-0.44	0.43	-0.37	-1.24
**sll0427**	PSII manganese-stabilizing polypeptide (psbO)	-0.47	-0.07	-0.34	-0.74
**sml0008**	PSI subunit IX (psaJ)	-0.49	0.14	-0.27	-0.90
**ssr0390**	PSI reaction center subunit X (psaK1)	-0.51	0.11	-0.42	-1.03
**sll0199**	plastocyanin (petE)	-0.78	-1.83	0.01	1.06
**slr2051**	phycobilisome rod-core linker polypeptide (cpcG)	-0.85	0.17	-0.50	-1.53
**sll0849**	PSII reaction center D2 (psbD)	-0.91	0.39	-0.11	-1.41
**slr1655**	PSI subunit XI (psaL)	-0.93	0.21	-0.27	-1.41
**sll1031**	carbon dioxide concentrating mechanism (ccmM)	-0.94	-0.08	-0.36	-1.23
**sll1322**	ATP synthase A chain of CF(0) (atpB)	-1.00	-0.24	-0.03	-0.79
**sll1327**	ATP synthase gamma chain (atpG)	-1.02	-0.41	-0.31	-0.92
**sll0819**	PSI reaction center subunit III (psaF)	-1.10	0.31	-0.49	-1.90
**ssl2615**	ATP synthase C chain of CF(0) (atpE)	-1.16	-0.22	0.20	-0.74
**sll1029**	carbon dioxide concentrating mechanism (ccmK)	-1.23	-0.10	-0.48	-1.61
**sll0851**	PSII CP43 protein (psbC)	-1.35	0.26	-0.16	-1.77
**slr0906**	PSII core light harvesting protein (psbB)	-1.39	0.34	-0.25	-1.98
**slr1329**	ATP synthase beta subunit (atpD)	-1.44	-0.55	-0.34	-1.23
**slr1835**	PSI P700 chlorophyll a apoprotein A2 (PsaB)	-1.57	0.33	-0.11	-2.01
**slr1834**	PSI P700 chlorophyll a apoprotein A1 (PsaA)	-1.57	0.31	-0.07	-1.96
**sll1326**	ATP synthase alpha chain (atpA)	-1.59	-0.25	-0.33	-1.67
**sll1580**	phycobilisome rod linker polypeptide (cpcC1)	-1.60	0.28	-0.30	-2.18
**sll1578**	phycocyanin alpha subunit	-1.62	0.11	-0.32	-2.04
**sll1324**	ATP synthase B chain (subunit I) of CF(0) (atpF)	-1.62	-0.31	-0.01	-1.31
**slr2067**	allophycocyanin alpha subunit (apcA)	-1.65	0.18	-0.12	-1.94
**sll1577**	phycocyanin beta subunit (cpcB)	-1.70	0.06	-0.17	-1.93
**slr0335**	phycobilisome core-membrane linker polypeptide (apcE)	-1.71	0.08	-0.52	-2.31
**slr1986**	allophycocyanin beta subunit (apcB)	-1.72	0.12	-0.10	-1.94
**ssl3093**	phycobilisome small rod linker polypeptide (cpcD)	-1.79	0.05	-0.39	-2.23
**ssr3383**	phycobilisome small core linker polypeptide (apcC)	-1.97	-0.01	-0.42	-2.38
**sll1579**	phycobilisome rod linker polypeptide (cpcC2)	-2.02	0.25	-0.34	-2.62

Gene expression changes were compared in cells of *Synechocystis* WT and ∆*sliP4* mutant after 48 h of HL (250 μmol photons m^−2^ s^−1^) exposure compared to gene expression at growth light (0 h, 50 μmol photons m^−2^ s^−1^). The locus IDs are given according to previous work (Kaneko et al. 1996, Kaneko et al. 2003), followed by the description of the gene products and four columns with the respective pairwise gene expression changes (log_2_FC) as indicated.

### Proteome and transcriptome changes align

A proteome analysis was performed for both strains before and after 48 h of exposure to elevated light levels. In total, 2 198 proteins of the 3 510 proteins of *Synechocystis* in our sequence database were identified in both strains (Supplementary Table S3), corresponding to a proteome coverage of 62.6%.

Many changes in the proteome corresponded to changes also observed in the transcriptome. The WT and mutant ∆*sliP4* exhibited similar decreases in phycobilisome, PSI, and PSII subunits, with marginally higher decreases observed in the mutant (Table 3). In contrast, the NDH subunits exhibited divergent responses in the mutant upon exposure to 48 h of HL intensity; the subunit NdhD1 (Slr0331) was increased, while NdhK1 (Slr1280) and NdhG (Sll0521) were decreased (Table 3). Interestingly, the NdhD1 (Slr0331) subunit was already found to be more abundant in the mutant before HL treatment at time 0 h. Of particular interest were the proteins PsbL (Smr0007) and PsaC (Sll0563), which exhibited the most significant decreases in abundance between WT and ∆*sliP4* after 48 h under HL. For the PsbL protein, the levels were quadrupled in the WT after exposure to HL, whereas the opposite effect was observed in the mutant, i.e. decreasing levels. In contrast, several proteins associated with the CRISPR2-Cas system (Sll7063, Sll7064, Sll7065 and Sll7066) were more abundant in the mutant growing under growth light conditions and under HL in comparison with the WT; however, this system was found to have its levels reduced by up to sevenfold after 48 h of exposure to HL in the mutant (Table 3).

**Table 3:** Proteomics after long-term high light (HL) treatment.

protein	protein description	∆*sliP4* 48 h vs 0h	WT 48 h vs 0h	∆*sliP4* 0 h vs WT 0h	∆*sliP4* 48 h vs WT 48h
**Sll7066**	CRISPR2-Cas II (cas7-cas5-cas11)	-2.40	-1.91	2.38	1.90
**Sll7063**	CRISPR2-Cas II (cas7 with insertion)	-2.63	-1.68	2.62	1.66
**Sll7065**	CRISPR2-Cas II (cas7-2x)	-2.66	-0.79	3.46	1.59
**Slr1281**	NAD(P)H-quinone oxidoreductase subunit J (NdhJ)	0.88	-0.82	-0.41	1.29
**Sll1262**	NAD(P)H-quinone oxidoreductase subunit N (NdhN)	0.85	-0.24	-0.31	0.78
**Slr0884**	glyceraldehyde-3-phosphate dehydrogenase 1 (GapDH1)	0.53	-0.13	0.10	0.76
**Sll7064**	CRISPR2-Cas II (csx19)	-1.72	-0.71	1.69	0.69
**Sll5042**	probable sulfotransferase (xssA)	-0.98	-0.67	0.81	0.50
**Sll0020**	ferredoxin (PetF)	1.01	0.73	0.19	0.47
**Sll1323**	ATP synthase subunit b' (AtpF2)	-0.72	-1.46	-0.29	0.45
**Slr0331**	NAD(P)H-quinone oxidoreductase chain 4–1 (NdhD1)	1.05	2.85	2.22	0.41
**Slr1280**	NAD(P)H-quinone oxidoreductase subunit K1 (NdhK1)	-0.28	-0.97	-0.29	0.41
**Sll0521**	NAD(P)H-quinone oxidoreductase chain 6 (NdhG)	-0.71	-1.05	-0.11	0.22
**Sll1398**	PSII reaction center (Psb28)	-1.50	-1.60	0.04	0.14
**Sll1452**	nitrate/nitrite transport system ATP-binding (NrtC)	0.90	0.89	0.09	0.11
**Slr1330**	ATP synthase epsilon chain F1 (AtpC)	-0.82	-0.55	0.37	0.09
**Sll0199**	plastocyanin (PetE)	-1.79	-0.72	1.02	-0.05
**Sll1577**	phycocyanin beta subunit (CpcB)	-0.79	-0.57	0.10	-0.12
**Slr1655**	PSI reaction center subunit XI (PsaL)	-0.79	-0.45	0.15	-0.19
**Ssr2831**	PSI reaction center subunit IV (PsaE)	-1.48	-0.72	0.57	-0.20
**Sll1194**	PSII 12 kDa extrinsic protein (PsbU)	-1.08	-0.63	0.17	-0.28
**Sll0629**	PSI reaction center subunit 2 (PsaK)	0.00	0.94	0.66	-0.28
**Sll0427**	PSII manganese-stabilizing polypeptide (PsbO)	-0.92	-0.56	-0.06	-0.42
**Slr1834**	PSI P700 chlorophyll a apoprotein A1 (PsaA)	-0.79	-0.15	0.12	-0.52
**Sll1580**	phycobilisome 32.1 kDa linker polypeptide, rod 1 (CpcC1)	-1.10	-0.27	0.06	-0.77
**Ssl2598**	PSII reaction center protein H (PsbH)	-0.09	1.08	0.29	-0.88
**Slr1835**	PSI P700 chlorophyll a apoprotein A2 (PsaB)	-0.55	-0.10	-0.43	-0.88
**Sll1579**	phycobilisome 32.1 kDa linker polypeptide, rod 2 (CpcC2)	-1.26	-0.77	-0.45	-0.95
**Ssl0563**	PSI iron-sulfur center (PsaC)	-1.60	-0.32	-0.31	-1.60
**Smr0007**	PSII reaction center protein L (PsbL)	-1.79	1.97	0.23	-3.53

Protein abundances in the *Synechocystis* WT and the ∆*sliP4* mutant after 48 h of HL (250 μmol photons m^−2^ s^−1^) exposure were related to protein amounts at growth light (0 h, 50 μmol photons m^−2^ s^−1^). Furthermore, relative protein abundances in both strains are given at growth light (0 h) or 48 h HL treatment, The locus IDs are given according to previous work (Kaneko et al. 1996, Kaneko et al. 2003), followed by the description of the proteins and four columns with the respective pairwise gene expression changes (log_2_FC) as indicated.

### Metabolome analysis indicates an enhanced photorespiration rate

**Figure 3:**
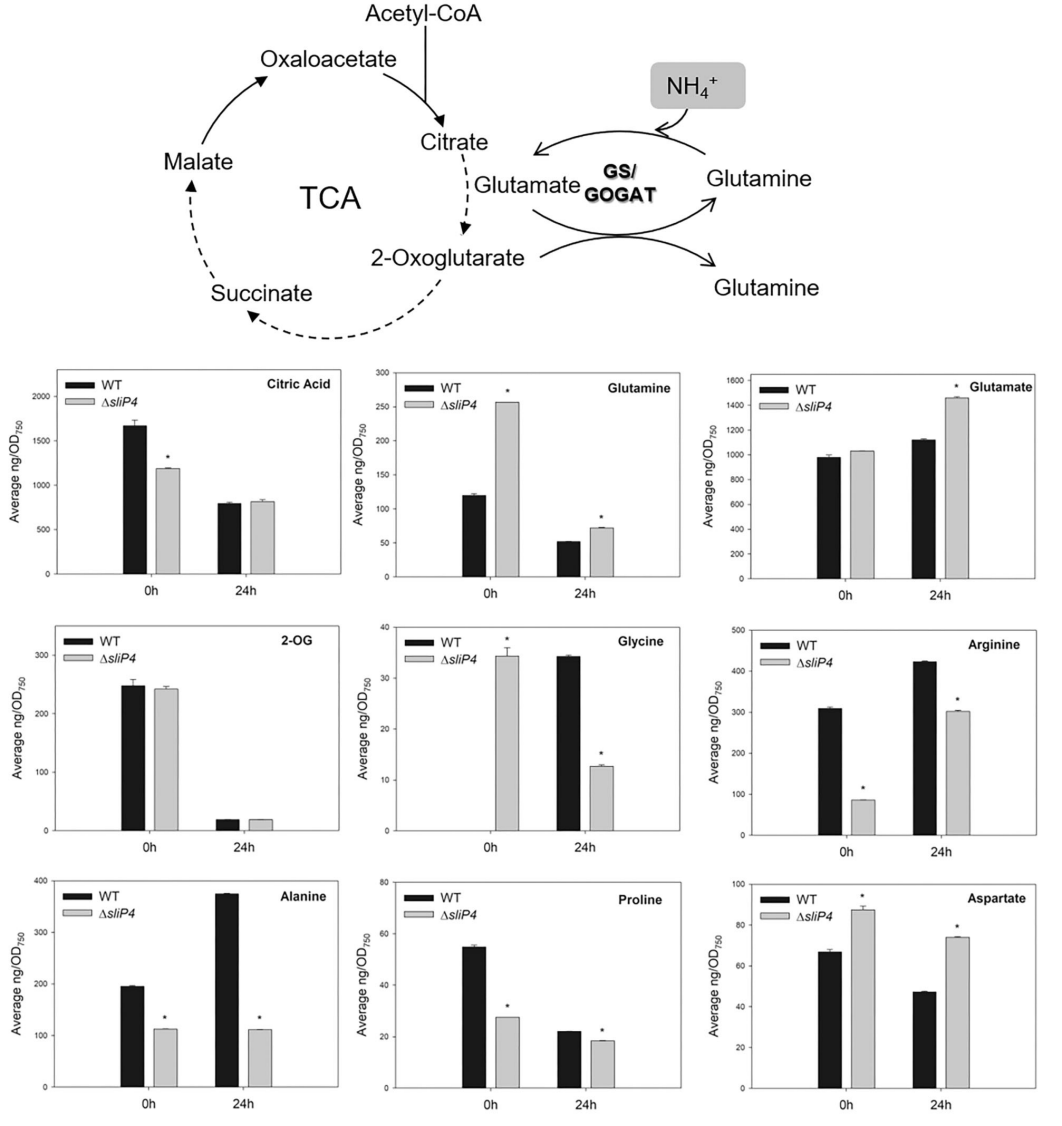
Changes in metabolite abundances after long-term high light (HL) treatment. Metabolite changes were compared in cells of the WT and the ∆*sliP4* mutant after 24 h of HL (250 μmol photons m^−2^ s^−1^) exposure compared to metabolite amounts at growth light (0 h, 50 μmol photons m^−2^ s^−1^). Metabolites of the central carbon and nitrogen metabolism were quantified by LC-MS/MS in extracts of the mutant ∆*sliP4* (grey columns) or the WT (black columns) of *Synechocystis*. The relative metabolite abundance can be found in the Supplementary Table S4. Asterisks indicate statistically significant differences (Student’s t-test, *P*-value < 0.01) (2-OG–2-oxoglutarate).

The ∆*sliP4* mutant develops a less-pigmented phenotype following 48 h of exposure to HL (Alvarenga-Lucius et al. 2023). In order to analyse these changes in more detail, a targeted metabolomics approach was employed utilising LC-MS/MS analysis before and after 24 h of cultivation under HL conditions, thereby providing a comprehensive overview of the steady-state levels of 20 cellular metabolites (Supplementary Table S4), including most amino acids. The results obtained revealed that the majority of the metabolites exhibited a comparable pattern in the WT and the ∆*sliP4* mutant after 24 h of HL exposure, characterised by a decline in valine, glutamine, proline, aspartate, 2-oxoglutarate (2-OG), lactic acid, citric acid, lysine, and an increase in arginine and glutamate levels (Fig. 3). In contrast, threonine, alanine and leucine levels increased in the WT, yet remained unaltered in the mutant. Furthermore, the TCA cycle intermediates citric acid and 2-OG showed lowered values in cells of the mutant as well the WT. Glycine exhibited the most significant differences between the two strains (Fig. 3). In the WT, glycine levels increased following exposure to HL, indicating an enhanced photorespiration rate. However, the mutant exhibited elevated glycine levels already at time 0 h before HL treatment, subsequently leading to an unexpected reduction in its levels after the cells were transferred to HL. The levels of serine remained similar after HL treatment in WT cells but decreased like glycine in cells of mutant ∆*sliP4* (Supplementary Table S4). The resulting different glycine/serine ratios may indicate different regulation of serine-hydroxymethyl transferase activities in both strains upon HL stress.

### Soluble and insoluble carbohydrate accumulation

**Figure 4:**
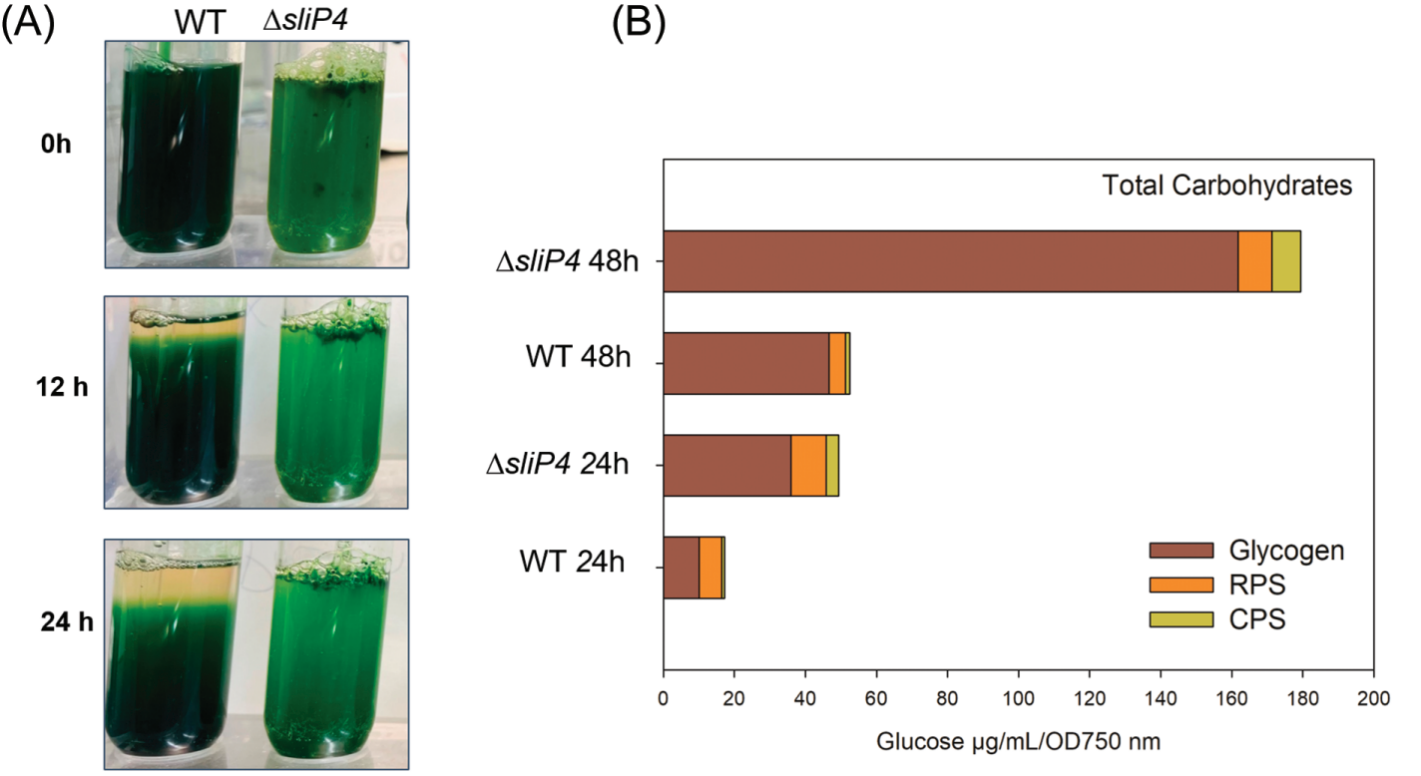
Floating behavior and accumulation of polysaccharides after long-term high light (HL) treatment. A. The sedimentation or floating of cells of *Synechocystis* WT and ∆*sliP4* mutant were compared in standing cultures according to Maeda et al. (2021). After cell exposure to HL for 24 h under 5% CO_2_ aeration, cells were left standing without aeration for 12 h and 24 h to check the sedimentation and formation of aggregates. B. Amounts of polysaccharides in WT and ∆*sliP4* mutant after of HL exposure for 24 or 48 h. RPS—Released EPS, CPS—Cell-bound EPS.

The gene expression analysis revealed a marked increase in the expression of synechan genes (Table 1), which are responsible for the synthesis of a specific class of EPS (Maeda et al. 2021). Therefore, we conducted experiments to verify that this change has some physiological meaning. First, cells exposed to 48 h of HL were subjected to a precipitation test. In this experiment, the cells were left to stand without movement or aeration under continuous dim light as done by Maeda et al. (2021), which revealed that synechan accumulation prevented fast sedimentation of *Synechocystis*. In our case, the WT cells precipitated slowly within 48 h towards the bottom of the flask, while ∆*sliP4* mutant cells remained more homogenously distributed in the medium (Fig. 4A). Furthermore, a proportion of cells from the mutant culture remained in a floating, aggregated formation. Then, we determined high molecular mass carbohydrates in the two strains. The floating behavior of the ∆*sliP4* mutant can be correlated with the higher amounts of released EPS (RPS) and cell-bound EPS (CPS) accumulated by this strain (Fig. 4B). It is noteworthy that the mutant also contained three times more glycogen than the WT, both prior to and following exposure to HL. This elevated level was sustained, with concentrations above 160 µg glucose units mL^−1^ OD_750nm_^−1^. The higher amounts of glycogen might indicate a higher flux of carbon in the direction of gluconeogenesis under HL, which is corresponding to lower carbon pools in the TCA cycle, for example citric acid (see. Fig. 3).

## Discussion

It has been demonstrated that exposure to elevated light intensities results in an increase in the excitation pressure on the photosystems, eventually leading to an overproduction of ROS. The primary target of ROS action is the D1 protein in the PSII. As outlined above, redirection of excitation energy among the photosystems due to activation of CEF and state transitions are among the HL acclimation strategies in cyanobacteria. Recently, we reported that in the absence of the small protein SliP4, *Synechocysti*s cells exhibit reduced CEF and state transition compared to WT resulting in impaired growth upon HL stress (Alvarenga-Lucius et al. 2023). This phenomenon most likely resulted from deficiencies in abilities to form photosynthetic supercomplexes, i.e. PSI/NDH1 association for CEF and PSI/PSII association for state transitions, which eventually resulted in redox imbalances. However, we could not rule out that the small SliP4 protein might be somehow involved in the process of HL stress signaling.

In the present study we analyzed the system-wide impact of SliP4 deficiency on HL acclimation combining different omics technologies. Basically, our results show that the mutant ∆*sliP4* can activate the known set of HL-induced genes and proteins like WT cell (see Tables S1-S3). As reported before (e.g. Hihara et al. 2001), genes encoding several HliPs or alternative forms of the D1 protein were expressed at higher levels, whereas genes for the light-harvesting phycobilisome antennae or PSI became decreased. These findings make it unlikely that SliP4 plays an important role in sensing or transmitting of HL-related signals in the Hik33/RpaB network. Due to the overlap between the transcriptome and proteome in many of these genes it is reasonable to assume that these changes are caused by transcriptional regulation. However, posttranscriptional effects for example by the action of regulatory sRNAs (e.g. *psrR1*) or on different protein stability cannot be excluded.

Among the genes and proteins that were differentially regulated in ∆*sliP4* compared to WT, we noticed altered levels of mRNA and protein abundances for specific NDH1 and photosystem subunits. The specific effect on their expression supports the assumption that SliP4 is mainly involved in the association of photosynthetic complexes under HL conditions, i.e. the small protein plays rather a structural than a regulatory role. After exposure to elevated levels of light, the WT appears to have acclimated to the HL conditions, because it is characterized by higher levels of PSII reaction center protein L (PsbL) and NAD(P)H-quinone oxidoreductase chain 4–1 and nitrate importer subunits such as NrtC after 48 h of HL treatment. In contrast, the mutant ∆*sliP4* exhibited a general decrease in protein content corresponding to the transiently lowered growth, except for a select number of NDH subunits (NdhD1, NdhJ, NdhN), ferredoxin (PetF), and PSI assembly protein (Ycf3). Interestingly, downregulation of genes for phycobilisome and upregulation of genes for NDH1 subunits were observed in the ∆*sliP4* mutant prior to exposure to HL levels, suggesting that even under growth light conditions the SliP4 protein is involved in proper excitation energy distribution between the different photosynthetic complexes.

An unexpected finding was the differential regulation of several genes known to be related to nitrogen imbalances (e.g. Krauspe et al. 2021). Before exposing cells to the HL treatment (0 h), the ∆*sliP4* mutant exhibited reduced expression of genes encoding nitrogen transporters and the regulatory PII protein (GlnB), which is crucial for sensing and responding to changed C/N ratios in cyanobacteria (Forchhammer et al. 2022). The expression of these genes was strongly elevated at 30 min after HL shift. This finding suggests that the cells were attempting to restore C/N balance. It is known that nitrogen assimilation, especially nitrite reductase (Kraus et al. 2024) and glutamate synthetase use reduced ferredoxin from the PSI and can serve as an electron sink to mitigate redox imbalances caused by defective CEF. Signs for an imbalance in the C/N ratio were also found in our metabolome analyses, in which we observed significant changes in amino acids such as aspartate, arginine and glutamine between ∆*sliP4* mutant and WT at the time points 0 h (growth light) and 48 h of HL. However, the changes in the levels of 2-OG, which serves as a signal metabolite for the C/N ratio and changes the PII structure (Forchhammer et al. 2022), were similar in WT and ∆*sliP4* cells. In both cases, the 2-OG level declined after HL treatment indicating a high C/N status within the cells after longer HL exposure. The similar amounts of 2-OG in the two strains rule out that this metabolite is responsible for the changed expression of the genes involved in N-metabolism. However, in addition to 2-OG the PII protein also measures the cellular energy status via ATP binding (Forchhammer et al. 2022), which is most likely different between WT and mutant ∆*sliP4* under HL stress.

Moreover, the ∆*sliP4* mutant proteomic data demonstrated an induction of glyceraldehyde 3-phosphate dehydrogenase 1 (GapDH1), whereas the amounts of GapDH2 decreased non-significantly following prolonged exposure to HL. Previous studies revealed that GapDH1 is specifically involved in carbon catabolism, while GapDH2 serves for the anabolic Calvin cycle (Koksharova et al. 1998, Lucius and Hagemann 2024). Their differential expression is another sign of a changed carbon metabolism, which is also visible in the elevated glycogen storage of the mutant ∆*sliP4*. Glycogen is over-accumulated in cyanobacterial cells under nitrogen limitation due to the inhibition of phosphoglycerate mutase (Orthwein et al. 2021), or CO_2_ excess (Eisenhut et al. 2008). Furthermore, the unexpected induction of synechan genes in ∆*sliP4* cells indicated a higher EPS synthesis in the mutant, which was verified by measuring EPS amounts. It is hypothesized that EPSs could also function as a form of carbon storage, thereby acting in addition or alternatively to glycogen as a sink for excess carbon and energy in cyanobacteria under diverse growth limiting conditions to prevent over-reduction of the photosynthetic electron transport (Santos et al. 2021, Madsen et al. 2023). The upregulation of synechan genes was particularly pronounced at 30 min after HL shift in the mutant ∆*sliP4*, which may point to a specific compensating reaction to deal with HL treatment in the absence of this small protein. It has been shown that the EPS synechan is especially involved in the aggregation and floating of *Synechocystis* under certain conditions (Maeda et al. 2021). Similarly, the higher expression of synechan genes correlated well with increased floating and aggregation of ∆*sliP4* cells compared to WT (see Fig. 4A). This decreases the light exposure of a single cell, thereby improving the HL tolerance. A similar behavior was recently found in an approach using adaptive laboratory evolution to generate *Synechocystis* variants with improved light tolerance (Dann et al. 2021). Hence, the enhanced synechan expression and accumulation is not only part of the changed carbon flux but also a specific compensatory reaction to deal with HL treatment in the absence of SliP4. This strategy allowed the less pigmented cells of the ∆*sliP4* mutant to resume growth and pigmentation under HL conditions eventually, as shown previously by Alvarenga-Lucius et al. (2023).

Collectively, our data rule out that the small SliP4 protein plays an important role in the sensing of HL stress, because basically similar transcriptomic and proteomic responses regarding genes/proteins with assigned functions to acclimate towards HL stress were observed in cells of the ∆*sliP4* mutant and the WT after HL treatment. Corresponding to the previously proposed function of SliP4 in the reorganization of photosynthetic complexes under HL conditions to support elevated cyclic electron transport and state transition (Alvarenga-Lucius et al. 2023), distinct expression changes were found particularly for subunits of NDH1 complex and PSI. Remarkably, some of these changes were observed already in cells grown at standard light, which provided first hints that SliP4 is not only involved in the proper organization of the photosynthetic complexes under HL, but also standard light conditions. Finally, the absence of SliP4 was compensated by a new avoidance strategy due to the activation of a population-level response to cope with HL conditions. Our transcriptome analysis identified the specific induction of genes of the *xss*-gene cluster, which is responsible for the synthesis of the EPS synechan (Maeda et al. 2021). While the detailed regulatory axis from sensing light stress to synechan induction was beyond the scope of this work, it is an interesting topic for future research. However, our physiological experiments verified that this elevated gene expression indeed resulted in aggregate formation of ∆*sliP4* mutant cells, thereby decreasing the light stress for single cells in the absence of other HL acclimation responses such as elevated cyclic electron transport and state transition.

## Supplementary Material

uqag003_Supplemental_File

## Data Availability

Mass spectrometry raw data were deposited at the ProteomeXchange (Deutsch et al. 2023) Consortium (http://proteomecentral.proteomexchange.org) via the PRIDE partner repository (Perez-Riverol et al. 2025) under the identifier PXD065318. Reviewer can access the data after log in to the PRIDE website using the following details: Project accession: PXD065318; Token: AyoabvljK5iE. Alternatively, reviewer can access the dataset by logging in to the PRIDE website using the following account details: Username: reviewer_pxd065318@ebi.ac.uk; Password: AO6lxUjQtQLV. The microarray data have been deposited in the GEO database under the accession number GSE302282.
